# A Limbo for the Queer

**Published:** 1884-10

**Authors:** Ben. H. Pratt


### A LIMBO FOR THE QUEER.

There are many strangely acting people in the world, who display so much of method in their strangeness that one cannot call them insane, and yet one feels that they are not wholly sane. They are regarded as *compos mentis*, for all the ordinary intents and purposes of life, but are they strictly so? It is, on the contrary, more than probable, that there are more insane people outside of our lunatic asylums and insane hospitals than there are within them. These are the queer people whom we meet in everyday life, and of whom we are continually saying, when their idiosyncrasies come up for discussion, or when we have occasion to refer to them at all, "it's only his way," or, "well, you know she is sort of queer, anyway," or, "he or she is all right when you know how to take them;" and when we come to think over these people they really seem to constitute the larger portion of our acquaintance. It is almost impossible for one to recall the name of a single acquaintance who is not more or less a trifle queer at times.

It seems to me that there is not being given to mental disorders that intelligent study which the age is giving to other and less important matters. We have not progressed in this direction in the same ratio that characterizes our advancement in other realms of human thought. In fact we are behind our quite remote ancestors in this particular. Look at that quaint but most philosophical of treatises by the genial Burton. His "Anatomie of Melancholie" is one of the most delicious specimens of English essay writing. What rare philosophy! what splendid truth! how grand his reasoning! how uplifting his thought! how incontrovertible his facts! how logical his argument! how entertaining he makes his science of the mind! how incisive his expression! how fascinating, from preface to finis, the whole work! But Burton is not read at this day. He, like many other of our dead and gone thinkers, has become passe, or is forgotten, or, alas! few know that such as he ever lived and wrote and comforted and informed his contemporaries.

No, the philosophy of the mind is not a subject of contemplation now. But the time is coming when it will be. It will come to pass some of these days that mankind will awake to the necessity of a full and exhaustive consideration of this great subject, and it will be because such consideration will have become a necessity. That time is not a great way off. In fact it would have exhibited wisdom on the part of mankind had they begun the study of slight departures from normality in minds long ago. Look at the terrible array of murders, of suicides, of strange things done, as they appear to us every day in the columns of the newspapers. Nine-tenths of all these are the acts of men and women who are insane and have been insane for years, and yet were not regarded as sufficiently insane to become the inmates of such institutions as we now have for the treatment and confinement and protection of the insane—or whom we call insane.

The term insanity does not now cover a broad enough field. It does not embrace a tithe of the insane among us. There is no place within the class of insane for the queer, the odd, the strange people we know, and there is no means of treating and no disposition to treat the incipiently insane among us. The opportunity must come, sooner or later, and if late it will be forced upon us. There are physicians of the mind, but they are occupied with chronic cases. These are specialists whosé lot is to be pitied, for they never have half a chance to exercise their art, knack or skill upon a curable case. The physician of the mind does not get to his patient, nor does his patient come to him, until he is really beyond the probability of being even helped, much less cured. He is shown no acute cases. He cannot treat a patient until he is so far gone that only a species of miracle can save him. The incipient stage of the mind's ailment is let alone. A man or woman must go clear crazy before he or she is treated for a mental disorder.

Suppose we carried this same sort of treatment into ordinary medical practice. Suppose we never allowed a man or woman who is visibly losing health and strength, to be treated for their malady until they were down sick physically; until they were helplessly laid up, and dissolution were imminent. Suppose we waited until our physical energy was so impaired that we could not raise hand or foot, or speak above a breath, before we called a doctor. It is when people begin to lose their health, begin to feel out of sorts, begin to realize that something is wrong with the corporeal economy, that they call a doctor, and it is even then, sometimes, quite difficult to arrest the disease, even in its germ period. If we waited for our bodies to become wrecks, as we do wait for our minds to become so, before summoning skill in the treatment of the disease, who could live out half his days? We must grapple with the mind before the mind is in its dying stage.

Now, why not use the same reasoning in the treatment of mental and physical diseases? Why do we allow our queer friends to go on in their queerness, getting queerer and queerer every year or every month or every week, without so much as an intimation to them that they are entirely too brilliant, or entirely too stupid, or too quick or too mean, or too unreasonable, or too lopsided, or too something or other, as the case may be, for a sane person. We all recognize the characteristics of complete sanity. There is a balance in the phenomena that is unmistakable. Any marked departure from these without due cause reveals insanity of which queerness is but the initial stage. But we don't act upon our knowledge. We have convictions on the subject that we don't put into any practical working. We simply admit that these people are queer, put up with their freaks, humor their oddities, avoid exciting them, perhaps, and make ourselves pretty much eternally miserable.

Now why should nine-tenths of mankind submit to the whims of the other tenth, humor their contemptible notions, be continually on the rack for fear of doing or saying or looking something that will arouse the anger or the sulks or the fretfulness or some other unpleasant demonstration in this fraction of the race. Men and women were made to live happily together. It was never intended that mankind were to go among each other continually on the alert lest one may say or do something, however unintentionally, that will throw somebody else into a spasm of disagreeableness or worse. Some people get along without any trouble to speak of. We call such, people having good common sense. That's the whole secret of it. People without common sense are insane, in a measure. We have a name for these lately. They are called cranks. It is too mild a term, but there doesn't seem to be any better, just yet. Cranks are insane. Perhaps all cranks are not dangerous, but they are none the less destitute of complete, healthy minds.

There should be institutions for the queer, the odd, the cranky. As before said, the time is coming when there will be physicians of the mind outside of the asylums and hospitals for the confirmed insane. Psychology has got to make some strides toward the practical. The time is approaching when queer people will not be humored but will be treated as mentally ill, and subjected to a treatment in accordance with the symptoms and the diagnosis. Just as we now go to a doctor for a strange feeling in the head or back or bowels or elsewhere, and don't wait until we are all broken down in health, so, by and by, queer people will go or be sent or be taken to the doctor of the mind just as soon as they exhibit symptoms of chronic queerness. It isn't in reason that disease—and here disease is plainly apparent—must be allowed to get the mastery of a man or woman before it is taken in hand.

It is because our insane are not taken in hand during the earlier stages of insanity that we have so many in our asylums. There should be some intermediary institutions, that will bear the same relations to our insane asylums that ideal reformatories bear to our State prisons, only they should be conducted upon the principle that cure is certain, and not merely probable. It is becoming more and more a fixed idea in the minds of many that the proportion of confirmedly insane people need not be at all considerable if maladies of the mind are taken in season and judiciously and intelligently treated. Of course there are some men and women who lose their minds suddenly, for cause, and become raving maniacs in a night, in an hour, or instantly; but these are the rare cases. The large proportion of crazy people in our asylums are those who for years have developed from queer to queerer, and without being treated have finally become incurable maniacs.

Such peculiar manifestations of mind as we see about us every day demand attention, and serious attention. A little prevention is worth an indefinite amount of cure. Before men and women reach the stage we now term insane, and while in the queer state, they can be treated without giving that offense, and without meeting that resistance which are such common accompaniments of the treatment which we now prescribe for the insane. When we get along a little further in this study of insanity it will become evident to the student that the range of that term will be much wider, and will embrace all phases of queerness, oddness, and eccentricity. Much that we term idiosyncrasy will be found to be insanity, and many who are now regarded as sane but peculiar will be classed as milder cases of insanity. So many are insane without knowing it—so many are not recagnized as insane because they are not violent or dangerous—that society is to-day a mixture of sane and insane that will surprise men and women of the future, who will wonder how society ever existed in the condition it now tolerates. The symptoms of these unacknowledged insane are yearly becoming more alarming. They are held to be responsible beings, when they are wholly irresponsible, and a manifest injustice is being done them. Business, society, the home, the church, all are sustaining a vast deal of inconvenience, to say the least, while there are thousands of individuals who are every day suffering untold impositions at the hands of these same people whose queer ways demonstrate insanity that is no less such because it is barely tolerable and has as yet called for no legal restraining process.

There is certainly a great need of attention to this phase of undeveloped insanity. All that is asked is that diseases of the mind be accorded the same prompt treatment that is given to disorders physical. It is attention to our more trifling ailments that often wards off the more serious. The crochety, the queer, the odd traits of character exhibited by mankind are as sure indices of disordered minds as boils, head-aches, back-aches, bowel-gripes and fainting fits are indices of systemic derangement. But we doctor the latter at once, while the former are allowed to correct themselves if they may, and ten to one they don't meet with correction. On the contrary, the men or women in whom the indications of mental disorders exist are oftener encouraged in the combination and development of them than otherwise. There are too few mind doctors, and these few are not given half a chance. They don't get their patients until they are beyond the probabilities of cure, and because they are not then cured we lose faith in all mind healing treatment. We want mind cures that are not asylums for the mad alone. We want places where queer people can be cured of their queerness, sort of Limbos for the Queer—something between a retreat and an asylum—and about one-tenth of the human family need to be taken there and cared for right off, to the end that the remaining nine-tenths may get some comfort out of this life.

